# Role of stress and early-life stress in the pathogeny of inflammatory bowel disease

**DOI:** 10.3389/fnins.2024.1458918

**Published:** 2024-09-10

**Authors:** Bruno Bonaz, Valérie Sinniger, Sonia Pellissier

**Affiliations:** ^1^Université Grenoble Alpes, Service d’Hépato-Gastroentérologie, Grenoble Institut Neurosciences, Grenoble, France; ^2^Université Savoie Mont Blanc, Université Grenoble Alpes, LIP/PC2S, Chambéry, France

**Keywords:** autonomic nervous system, inflammatory bowel disease, early-life stress, stress, vagus nerve

## Abstract

Numerous preclinical and clinical studies have shown that stress is one of the main environmental factor playing a significant role in the pathogeny and life-course of bowel diseases. However, stressful events that occur early in life, even during the fetal life, leave different traces within the central nervous system, in area involved in stress response and autonomic network but also in emotion, cognition and memory regulation. Early-life stress can disrupt the prefrontal-amygdala circuit thus favoring an imbalance of the autonomic nervous system and the hypothalamic-pituitary adrenal axis, resulting in anxiety-like behaviors. The down regulation of vagus nerve and cholinergic anti-inflammatory pathway favors pro-inflammatory conditions. Recent data suggest that emotional abuse at early life are aggravating risk factors in inflammatory bowel disease. This review aims to unravel the mechanisms that explain the consequences of early life events and stress in the pathophysiology of inflammatory bowel disease and their mental co-morbidities. A review of therapeutic potential will also be covered.

## Introduction

Preclinical and clinical studies have shown that stress is one of the main environmental factor playing a significant role in the pathogeny and life-course of bowel diseases (Bonaz and Bernstein, 2013; Pellissier and Bonaz, 2017; Bernstein, 2017; Labanski et al., 2020). However, while today the place of stress, and particularly early life stress (ELS), is recognized in the pathogeny of functional digestive disorders such as irritable bowel syndrome (IBS) (Pellissier and Bonaz, 2017), this implication is still poorly studied in inflammatory bowel disease (IBD) and only recent clinical studies begin to bring some empiric arguments (Witges et al., 2019; Gnat et al., 2023; Minjoz et al., 2023). In this literature review, we propose to clarify the role of stress and ELS in IBD, in particular the mechanistic knowledge on the role and consequences of ELS in IBD, and propose therapeutic (either drug or non-drug) interventions. Previous reviews have focused mainly on the role of stress in IBD (Bonaz and Bernstein, 2013; Bernstein, 2017; Labanski et al., 2020) but not the role of ELS. Focusing on ELS as potential aggravating factors seems quite interesting because they may favor digestive, mental, and physical vulnerabilities in IBD patients (Minjoz et al., 2023).

**FIGURE 1:**
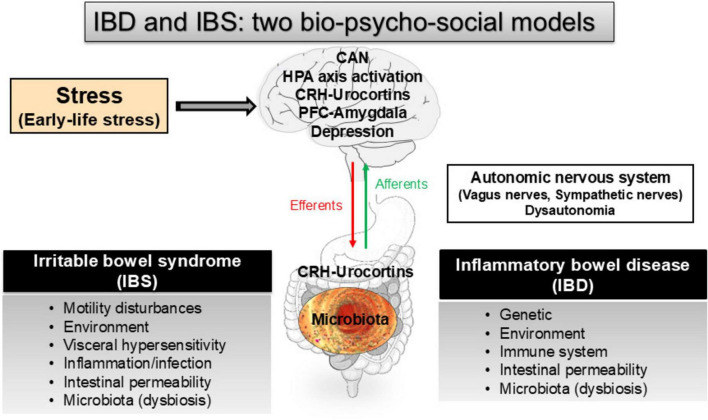
The role of stress and early-life stress along the gut-brain axis in irritable bowel syndrome (IBS) and inflammatory bowel disease (IBD), two bio-psycho-social models (see for review: Bonaz et al., 2012; Bonaz and Bernstein, 2013; Pellissier and Bonaz, 2017). CAN, central autonomic network; CRH, corticotrophin-releasing hormone; HPA, hypothalamic-pituitary adrenal; PFC, prefrontal cortex.

IBD, represented by Crohn’s disease (CD) and ulcerative colitis (UC), are organic diseases, mainly involving the small-bowel and/or recto-colon, typically starting early in life (15–30 years) and evolving by flares, characterized by abdominal pain, diarrhea, weight loss, and bloody stools (in particular in UC), alternating with variable periods of remission (Abraham and Cho, 2009). There is a rising worldwide incidence of IBD, with a particularly sharp increase in children (Sýkora et al., 2018). Rates are highest in North America and Europe, with rapid increases noted in developing nations adopting the Westernized environment. IBD have a significant impact on the patient quality of life (Knowles et al., 2018). The biopsychosocial model provides an understanding that results from a complex interaction of environmental (e.g., ELS experiences), psychological (e.g., depression, illness, anxiety, somatization), and biological factors (e.g., gut permeability, inflammation, dysbiosis) with bidirectional interactions of the brain-gut-axis (Bitton et al., 2008; Bonaz and Bernstein, 2013; Pellissier and Bonaz, 2017; Bernstein, 2017; Labanski et al., 2020; Dent et al., 2022; Figure 1). While ELS is well known to play a role in IBS pathogeny (Pellissier and Bonaz, 2017), by inducing perturbations of the functional GI tract integrity, such as motility, secretion, sensitivity, permeability, microbiota, and immunity, fewer data are available for IBD (Pellissier et al., 2010; Bonaz and Bernstein, 2013; Bernstein, 2017; Labanski et al., 2020). However, the prevalence of at least one adverse childhood experience is reported in ∼75% of IBD patients (Witges et al., 2019) and more recently in 53% of IBD patients (Minjoz et al., 2023). Gnat et al. (2023) also signalized that IBD patients report more sexual, disruptive, and violent traumas. Fuller-Thomson et al. (2015) reported that childhood physical and sexual abuse were related to UC, but not CD. Thus, one can hypothesize that ELS, as reported for IBS, may also favor and/or aggravate IBD course later in life.

In the present manuscript, we will develop stress concepts in their acute and chronic dimensions, as well as the hypothalamic-pituitary adrenal (HPA) axis and the autonomic nervous system (ANS), which are involved in the stress response (Figure 1). We will aim at underlining its effect on the brain and GI tract and discuss the possible stress involvement, and particularly ELS, in IBD. In the last part, we will explore the potential therapeutics either drug- and/or non-drug therapies that might be efficient to alleviate the part of stress effects on the expression of IBD.

## The stress concept

Stress is defined as the psychological and neurobiological response of the organism to an acute solicitation of the environment corresponding to the general syndrome of adaptation that permits to cope with the stressor to maintain homeostasis beyond changes. The neurobiological response, defined by Selye in 1936 (Selye, 1998), includes three stages: (i) an initial brief alarm reaction with adrenaline release puts the organism under tension and ready to react, followed by (ii) a prolonged period of resistance where cortisol release facilitates the body to cope with stress with an optimal use of energy and psychological resources, and (iii) a terminal stage that lead to adaptation and recovery in the case of eustress or a state of exhaustion and death in case of distress. The stress reaction is physiological, common to species, and essential for the body and adaptation. However, when the capacities of adaptation are outdated, psychologic, functional, metabolic and/or inflammatory disorders may appear. Indeed, excessive stress such as repeated exposures to stressful events with no possibility to recover, can result in cumulative biological changes (allostatic overload), that can be seen as a chronic stress state, altering adaptive mechanisms and resulting first in inefficient allostatic responses (excess or lack of epinephrine and/or cortisol non-adapted to a situation) and second in affected body systems (McEwen, 2000). Allostatic load and overload have adverse effects on development, immune response and regulation, metabolism, and other physiological functions (Chrousos, 2009).

### Stress and the hypothalamic-pituitary adrenal axis

The HPA axis is a major component of the neuro-endocrine-immune axis mediating stressor effects by regulating numerous physiological processes, such as metabolism, immune responses, and ANS (Chrousos, 2009). Early in life exposure to excess glucocorticoid (GC) hormones or environmental perturbations, such as maternal stressors, can disrupt the development of the HPA axis with deleterious consequences for the fetus later in life favoring abnormal physiological functions in adulthood and increasing the risk for adult diseases (Sheng et al., 2021). Circulating GCs bind both to GC and mineralocorticoid receptors involved in the negative feedback regulation of the HPA axis. Hypothalamic corticotrophin-releasing hormone (CRH), which controls adrenocorticotropic hormone secretion by the adenohypophysis, is released by parvocellular neurons of the paraventricular nucleus of the hypothalamus (PVH) and integrate neural or hormonal inputs leading to physiologic and metabolic responses to cope with the physiological changes following a stressor. These CRH neurons receive noradrenergic and adrenergic excitatory inputs from the nucleus of the solitary tract, locus coeruleus, and the ventrolateral medulla neurons (Cunningham et al., 1990). Projections from the median and dorsal raphe nuclei also strongly influence HPA responses to stress. Various limbic structures, such as the bed nucleus of the stria terminalis, hippocampus, prefrontal cortex (PFC), central nucleus of the amygdala, and lateral septum also target PVH neurons (Swanson and Sawchenko, 1980; McKlveen et al., 2015). These regions are involved in the emotional regulation of stress and are altered in case of ELS (Teicher et al., 2016).

### Abnormal programming of the fetal HPA axis

There is a critical period of development where the fetus is more sensitive to environmental influences, which significantly affects short- and long-term health, including maternal stress (Harris and Seckl, 2011). This leads to a fetal overexposure to GC, causing programming effects in the fetal HPA axis resulting in dysregulation of important physiological functions in adulthood. The placenta is a key player in such effect. Indeed, GC concentrations in maternal blood are higher than those of the fetus. 11β-hydroxysteroid dehydrogenase type 2 expression by the placenta oxidizes maternal corticosteroids into its inactive 11-keto derivatives, thus buffering the increased levels of maternal GC to the fetus. The levels of this enzyme are higher during early and mid-gestation than in late gestation to allow GC mediated fetal lung maturation (Harris and Seckl, 2011). The short- and long-term effects of GC maternal increases on the fetus depend on the length of exposure and period during development at which the insult occurs, the late gestation period being at the highest risk. Prenatal insults can impact ANS functioning in adult offspring exposed to fetal GC (Schlatterer and du Plessis, 2021). Such long-term consequences include cardiovascular and metabolic functions (Entringer et al., 2015).

In conclusion, abnormal development of the HPA axis can induce long-term alterations in neuropeptide and neurotransmitter synthesis within the central nervous system (CNS), as well as GC hormone synthesis in the periphery, potentially leading to a disruption in neuroendocrine, behavioral, autonomic, and metabolic functions in adulthood (Sheng et al., 2021).

### Stress and the autonomic nervous system

The ANS conveys sensory, chemical, mechanical, thermic, and nociceptive information from viscera, including the gut, through afferent fibers entering the CNS either at the level of the spinal cord, or the nucleus of the solitary tract in the medulla oblongata. The ANS is also composed of efferent fibers constituting the sympathetic (the splanchnic nerves) and the parasympathetic (vagus and pelvic nerves) branches to modulate the enteric nervous system and the local activities of the gut (Bonaz et al., 2017; Figure 1). The vagus nerve (VN) contains ∼80% of afferent fibers mediating sensorial inputs from the thoraco-abdominal viscera to the brain (Altschuler et al., 1989) except for the pelvic viscera for which information is mediated by the pelvic nerves to S2–S4 levels of the spinal cord with central projections like other spinal visceral afferents. The VN conveys mainly mechanical and chemical sensory information from the gut. While vagal afferents do not classically encode painful stimuli, they are able to modulate nociceptive processing at the levels of the spinal cord and the brain (Jänig et al., 2000; Saper, 2000). The VN is currently considered as the sixth sense of the body (Zagon, 2001). The sympathetic nerves contain 50% of visceral afferent fibers entering via spinal nerves (i.e., splanchnic nerves), at T5-L2 segments of the spinal cord, carrying information concerning temperature as well as nociceptive visceral inputs related to mechanical, chemical, or thermal stimulation through Aδ and C afferents, which will reach conscious perception. The sympathetic nerves also contain 50% of efferent fibers involved in the control of gastrointestinal (GI) motility and peripheral immune organs.

After reaching the CNS, visceral information is integrated in the central autonomic network (CAN) (Figure 1), a network of brain regions involved in the autonomic, endocrine, motor, and behavioral responses essential for survival (Benarroch, 1993) such as the anterior cingulate cortex, insular cortex, orbito-frontal and ventro-medial cortices, central nucleus of the amygdala, PVH, nucleus ambiguus, ventrolateral and ventromedial medulla. The CAN outputs are directly linked to positive and negative feedback loops governing both sympathetic and parasympathetic outputs on peripheral organs, including the immune system. Many of these reflex loops are unconscious (i.e., not perceived) but may become conscious (i.e., perceived as painful) in pathological conditions such as inflammation.

The VN has anti-inflammatory properties both through its afferents, activating the HPA axis, and efferents via the cholinergic anti-inflammatory pathway (CAP), putting the VN at the interface of the neuro–endocrine–immune axis (Bonaz et al., 2017, 2021a). The anti-inflammatory effect of the CAP is mediated through the release of acetylcholine (ACh) binding to α7 nicotinic cholinergic receptor of macrophages principally, thus inhibiting the release of tumor necrosis factor (TNF) (Wang et al., 2003). Another pathway, involving the spleen, and called the non-neuronal cholinergic pathway has been described by Tracey’s group (Rosas-Ballina et al., 2011). Some investigators also proposed alternative pathways involving the sympathetic nervous system (SNS), i.e., the greater splanchnic nerves (Martelli et al., 2016; Bonaz et al., 2021a).

**FIGURE 2:**
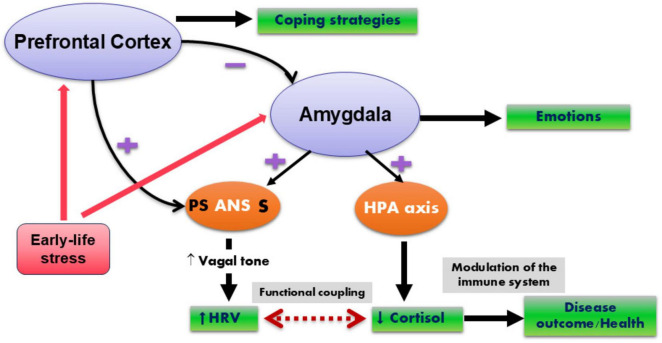
Interactions between the prefrontal cortex (PFC), amygdala, hypothalamic-pituitary adrenal (HPA) axis, and the autonomic nervous system (ANS), as well as the effects of early-life stress (ELS) on the PFC-amygdala complex. The PFC activates the vagus nerve while inhibiting the amygdala (which activates the HPA axis and the sympathetic nervous system). An imbalance between the prefrontal cortex and the amygdala in case of ELS results in an imbalance between the HPA axis and the ANS at the peripheral level (Pellissier et al., 2014). The lower the vagal tone, the less active the PFC will be, reflecting a shifting from a homeostatic state to a stress state. This allows the amygdala to be more active. HRV, heart rate variability; PS, parasympathetic (vagus nerve); S, sympathetic. +, activation; –, inhibition.

During the stress reaction, the parasympathetic system is blunted, as revealed by a decrease in vagal tone, reflecting the withdrawal of the cardiac vagal brake and allowing heartbeat acceleration and SNS dominance (Skora et al., 2022). This effect is mediated by central CRH and leads to the release of noradrenaline and adrenaline via locus coeruleus activation, two major coordinators of cardiovascular and respiratory, emotional, and cognitive adaptation to the stressful situation (Taché and Bonaz, 2007; Wood and Woods, 2007). Thus, CRH may function to inhibit exaggerated vagal activation that results in severe bradycardia or even vasovagal syncope. CRH1 receptor antagonists increase cardiac vagal and decrease sympathetic activity, implicating CRH1 as a therapeutic target for autonomic disturbances with an increased sympathetic activity, such as hypertension and coronary heart disease (Wood and Woods, 2007). Measuring vagal tone in correlation with cortisol represents a window opened on the homeostasis of the CAN (Pellissier and Bonaz, 2017). The resting vagal tone, as indexed by vagally-mediated heart rate variability (vm-HRV), is an “endophenotype” reflecting the balance of the autonomic network (Thayer and Lane, 2009). The outputs of the CAN are directly linked to vm-HRV, which has been proposed by Thayer and Lane (2007) as an indicator of CNS–ANS integration. Thus, cardiac vagal tone is an index of stress and stress vulnerability in mammals (Porges, 1995). In this integrative interplay, the functional coupling between low cortisol levels and high vagal tone at rest would reflect the tonic inhibition of the PFC on subcortical sympatho-excitatory circuits such as the amygdala (Bonaz et al., 2012; Figure 2).

The VN is a key component of the microbiota–gut–brain axis (Figure 1). It can sense microbiota metabolites through its afferents, transferring this intestinal information to the CNS, and generating an adapted or inappropriate response from the CNS to the intestine and the microbiota, according to a bidirectional communication (Bonaz et al., 2018; Kasarello et al., 2023). The VN, via the CAP, could modulate the intestinal microbiota by decreasing intestinal permeability and modulating local immunity (Bonaz et al., 2018; Bonaz, 2022). Consequently, a dysfunction of the VN could favor dysbiosis and intestinal inflammation thus participating to the pathogeny of IBD.

### Disorders of autonomic nervous system neurodevelopment

ANS regulatory capacity begins before birth as the sympathetic and parasympathetic activity contributes significantly to the fetus’ development. The VN is involved in many vital processes during fetal, perinatal, and postnatal life (Cerritelli et al., 2021). Pathologies can affect ANS development, altered ANS function can make newborns more vulnerable to those same pathologies, due to failure in activating efficient anti-inflammatory reflexes including the CAP (Mulkey and du Plessis, 2018; Bonaz et al., 2021a). ANS impairment may have consequences on the global development persisting all lifelong. ANS immaturity due to fetal or postnatal complications (e.g., maternal pathologies, birth difficulties) could then impair the whole brain development and, therefore, behavior, stress response, and mood regulation, with negative consequences-even serious neurological or psychological pathologies in infants, adolescent, and adult life (Mulkey and du Plessis, 2018, 2019). Several uncontrollable situations such as natural disasters, relational or financial problems, bereavement, and/or stressful daily hassles can threaten a woman’s life during her pregnancy. These situations increase the risk of impairment of fetal brain development resulting in emotional, behavioral, and/or cognitive problems in later life (Glover, 2015; Antonelli et al., 2022). A foeto-maternal pro-inflammatory state could interfere with the set point of the fetal vagal regulatory system through CAP dysregulation, thus impairing fetal intestinal permeability and integrity (Garzoni et al., 2013). Maternal mood disorders and depression seem to strongly impact the fetal neuroendocrine systems, in particular the amygdala (McEwen et al., 2016) and the hippocampus (Nemoda et al., 2015), involved in the CAN (Harrison et al., 2013; Thome et al., 2017). Moreover, maternal psychopathology influences the parasympathetic cardiac-vagal tone (as index through HRV) also after birth, showing a higher mean heart rate and lower vagal modulation in the new-born, and confirming the impact of this prenatal disorder on vagal neurodevelopment (Dierckx et al., 2009). Smoke and alcohol exposure can induce in premature new-borns higher sympathetic function, lower parasympathetic function, and less cardiac autonomic adaptability (Mulkey and du Plessis, 2019). Many diseases can impact the placental function, which in turn seems to have a role in regulating the neurodevelopment of the main factors that interact with the fetal VN, and the monitoring of placenta secretome could be a useful biomarker of diseases and potential developmental disruptions of the ANS in the fetus (Aplin et al., 2020).

## Stress in inflammatory bowel disease

The etiology of IBD involves a complex interaction between genetic, environmental, microbial factors, and the immune responses (Zhang and Li, 2014). Among environmental factors, a growing body of evidence argues for a role of stress, through the HPA axis, the limbic systems (e.g., the amygdala), and the ANS in the initiation and perpetuation of these diseases (Bonaz and Bernstein, 2013; Bernstein, 2017; Labanski et al., 2020; Figure 1). An imbalance of the ANS (dysautonomia) is one of the mechanisms underlying the role of stress in IBD pathogeny (Bonaz and Bernstein, 2013; Pellissier et al., 2014, 2010; Bernstein, 2017; Labanski et al., 2020). IBD and their emotional correlates bring the patients to develop a coping strategy, which in turn influences the course of their disease as reported in CD (Bitton et al., 2008). IBD is a biopsychosocial model (Bitton et al., 2008), as described for IBS (Mayer et al., 2001; Figure 1). There is evidence that psychological factors play a role in both the pathogeny and the course of IBD and how patients cope with IBD (Maunder et al., 2012). Bernstein et al. (2010) reported, in a population-based cohort of 552 IBD patients, that stress triggers symptomatic flares of IBD. Family stress was the most commonly reported, followed by work or school and financial stress. IBD patients with persistent inactive disease experienced no perceived stressful events compared with those with persistent active disease. Psychological factors, including occurrence of a major life event, high-perceived stress, and high negative mood during a previous 3-month period, were significantly associated with the subsequent occurrence of a flare. This study corroborates the growing evidence from animal as well as clinical studies that stressful events and high perceived stress (the individual’s view of their own level of demand relative to their resources), may contribute to relapse risk in IBD (Mawdsley et al., 2006; Bitton et al., 2003, 2008). Interestingly, on multivariate logistic regression analyses among several aggravating factors (as antibiotics for instance) only high-perceived stress was associated with an increased risk of flare. Being symptomatic may exacerbate or even incite stress (inflammatory, painful, cognitive and emotional stressors), whereas being stressed may trigger symptomatic disease as a real vicious circle. Jaghult et al. (2013) also reported an effect for high level of perceived stress in IBD patients in remission. Being exposed to “quite a lot” of stress, yielded an increase in risk for relapse during the forthcoming day. Targownik et al. (2015), from a population-based registry of IBD patients, found a strong relationship between perceived stress and GI symptoms but perceived stress was unrelated to concurrent intestinal inflammation. In a systematic review, Black et al. (2022) reported that psychological stress appears to precede IBD exacerbation.

Acute and chronic stress are associated with increases in C reactive protein and other inflammatory mediators (Steptoe et al., 2007; Del Giudice and Gangestad, 2018). Patients with UC compared with healthy controls, exhibited higher rectal mucosal proinflammatory response to stress with increased lipopolysaccharide-stimulated cytokines, leukocyte and natural killer cell counts, platelet activation, and production of reactive oxygen metabolites with reduced rectal mucosal blood flow (Mawdsley et al., 2006). In asymptomatic UC outpatients, the association of perceived stress with rectal mucosal abnormalities was strongly suggestive of a true link between psychological factors and UC activity (Levenstein et al., 1994). The relapse rates of IBD patients one and two years after the Great East Japan Earthquake significantly decreased by comparison with rates immediately after the earthquake. Only patients who had experienced the death of family members or friends were likely to need additional treatments (Miyazawa et al., 2018). However, some studies did not find a link between stress and IBD course (Li et al., 2004; Lerebours et al., 2007).

The pattern of expression of key stress regulators, such as members of the CRH family (CRH and Urocortins), in peripheral organs like the colon, plays a critical role. For example, the complete lack or low expression of the CRH2 receptor in the large intestine works positively in establishing, maintaining, and enhancing an inflammatory microenvironment in the organ (Baritaki et al., 2019). Lasconi et al. (2021) have recently revealed the role of the hypothalamus, the main source of CRH, in the genetic susceptibility to IBD. They propose that IBD-associated single-nucleotide polymorphisms alter the HPA axis and stress responses predisposing to and/or exacerbating this disease. They suggest that IBD genetic risk variants influence both general (e.g., immunologic and endocrine) and specific (e.g., symbiotic process) pathways in different cell types thus providing approaches to extending mechanistic analyses of genome-wide association study data for complex phenotypic traits.

Interestingly, Koren et al. (2021) reported that neuronal ensembles in the insular cortex are activated during a dextran sulfate sodium-induced colitis, a model mimicking UC. Reactivation of these neurons is sufficient to retrieve peripheral inflammation. These insular cortex neurons project to ANS control sites i.e., the dorsal motor nucleus of the VN and rostral ventrolateral medulla. Inhibition of the insular cortex alleviates inflammation during dextran sulfate sodium-induced colitis, thus revealing the potential of inhibiting insular cortex activity as a means of suppressing peripheral inflammation. The insular cortex is a site where bodily sensations (interoception), autonomic control and afferents from brain regions implicated in emotion processing, like the amygdala, converge (Bonaz et al., 2021b). The insular cortex is a major hub in emotion regulation and memory, multisensory integration, and valence processing (Gogolla, 2017). Thus, the insula cortex contains specific information about inflammation in the body and can regulate peripheral immune responses. As questioned by Gogolla (2021) in an editorial of the work of Koren et al. (2021): “Do stress and anxiety lead to reactivation, *per se*, or change how many cells are recruited into an immunological engram? Can stressful emotionally salient and/or early events known to activate the insular cortex trigger reactivation of immunological memories and elicit peripheral inflammation?” Thus, data by Koren et al. (2021) show clear evidence that the insular cortex communicates with the immune system, represents specific information about the inflammatory status in the body (interoception), and regulates peripheral immune responses, thus neurons in the brain recall gut inflammation (Brea and Veiga-Fernandes, 2022).

The different factors and pathways through which stress may play a deleterious role in IBD are the same than in IBS (Bonaz and Bernstein, 2013): (i) activation of mast cells, (ii) activation of the SNS and vagal inhibition, (iii) imbalance of the prefrontal-amygdala complex and the immune system, (iv) down-regulation of the hypothalamic CRHergic system, (v) role of the peripheral CRHergic system in inflammation (increased intestinal permeability), (vi) the microbiota brain-gut axis (intestinal barrier dysfunction and dysbiosis), (vii) aggravating effect of depression, and last but not least, (viii) aggravating effect of early life events.

## Early-life stress

ELS refers to the exposure to one or more stressful events during childhood that exceeds the child’s coping resources and leads to prolonged phases of stress response. ELS may include several types of situations, involving physical, sexual, emotional or verbal abuse, neglect, social deprivation, disasters or household dysfunction (including witnessing violence, criminal activity, parental separation, parental death or illness, poverty, addiction) (Brown et al., 2009).

At least 50% of the US adult population has experienced one or more ELS before the age of 18 years, but in clinical practice, ELS remain underrecognized (Schüssler-Fiorenza Rose et al., 2014). The US Centers for Disease Control and Prevention reported that of 100,000 adults studied across 25 states, 1 in 6 had experienced 4 or more types of adverse childhood experiences, and that 5 of the top 10 leading causes of death are associated with adverse childhood experiences. Preventing adverse childhood experiences could reduce the number of adults with depression by as much as 44% (Centers for Disease Control and Prevention, 2019). About 14.5% of women and 6.4% of men in France, that is approximately 5.5 million people, were faced with sexual violence before the age of 18 (Bajos et al., 2021). In a recent meta-analysis of 206 studies, representing 546,458 adult participants across 22 countries, Madigan et al. (2023) reported that the prevalence of ELS was 39.9% for no ELS, 22.4% for one ELS, 13.0% for two ELS, 8.7% for three ELS, and 16.1% for four or more ELS. Thus, 60% of adults reported at least one ELS, and one in six reported exposure to four or more ELS prior to age 18. Although these data suggest that ELS are common, the authors also found considerable disparities across the population. Especially, there was strong evidence of differences in the prevalence of 4+ ELS through samples with different sociodemographic, economic and health-related profiles (in particular, racial/ethnic features, household income, and history of a mental health condition or substance abuse/addiction).

ELS also includes prenatal maternal stress in humans caused by exposure to both severe stressors and milder forms of psychosocial or immune stressors during pregnancy. Maternal prenatal stress induces physiological signals such as high plasmatic cortisol levels that are subsequently transmitted to the fetus through the placenta, which may affect size at birth as well as long-term outcomes affecting later life (Monk et al., 2012; Godoy et al., 2021). Fetal malnutrition and fetal overexposure to GC or stress may explain the association between an adverse prenatal environment and postnatal health outcomes (Harris and Seckl, 2011).

ELS during the prenatal and postnatal periods affects the development of neural networks that influence brain function throughout life (Nishi, 2020). ELS can be predictive of future physical and mental health outcomes such as risk factors for chronic diseases (Campbell et al., 2016; Godoy et al., 2021). Indeed, ELS increases the risk of major psychiatric disorders, including anxiety, depression, and substance abuse disorders, but also lifelong risk for chronic diseases, including cardio-vascular diseases, obesity, diabetes, lung cancer, chronic pain, headaches, and immune-related diseases resulting in reduced longevity (Eskelinen and Ollonen, 2010; Norman et al., 2012; Godoy et al., 2021; Zhu et al., 2022; Bussières et al., 2023; Greenman et al., 2024; Koball et al., 2024; Hashim et al., 2024). In addition, detrimental effects of ELS may be transmitted from one generation to the next, thus extending the long-term ramifications of early adverse experiences and constituting intergenerational continuity in poor health outcomes (Moog et al., 2022). Consequences of ELS often depend on the type and number of adversity, the timing of the relevant exposure to stress and on the exposure to stress at certain periods of development that is the age at the time of the stress event. Indeed, maltreatment alters trajectories of brain development to affect sensory systems, network architecture and circuits involved in threat detection, emotional regulation and reward anticipation (Teicher et al., 2016). Early negative experiences alter plasticity processes during developmentally sensitive time windows and affect the regular functional interaction of cortical and subcortical neural networks involved in stress, cognition and emotion regulation, which in turn may promote a maladapted development with negative consequences on the mental and physical health of exposed individuals (Holz et al., 2023). Developmental programming refers to the concept of sensitive periods of plasticity, and assumes that a vulnerability occurs early in life, during periods when the brain and physiological systems are malleable, and that this vulnerability persists into adulthood and permanently shifts responsiveness to the environment beyond genetic factors (Heim et al., 2019). Brain regions with prolonged postnatal development are particularly vulnerable to the long-term effects of stress (Teicher et al., 2003). Associations between childhood trauma and reduced global and regional brain volumes using magnetic resonance imaging were reported, thus arguing for a lasting effect of childhood adversity on brain structure (Madden et al., 2023). ELS leads to structural, functional and epigenetic changes in a connected network of brain regions that is implicated in neuroendocrine control, autonomic regulation, vigilance, emotional regulation, and fear conditioning (Heim et al., 2019).

Among brain structures influenced by ELS, the amygdala, hippocampus, hypothalamus, and PFC, with dense expression of GC receptors (Lupien et al., 2009), are able to induce perturbations of the equilibrium between brain areas and neuroendocrine network of stress, namely the HPA axis and the ANS (Teicher et al., 2016; Figure 2). This can create an allostatic overload and a state of vulnerability (hyper or hyposensitivity; low grade tissue inflammation) favoring later development of chronic diseases with pain and inflammation such as IBS and IBD. Elevation of cortisol or inflammatory cytokines that occur as a consequence of ELS may exert neurotoxic effects on these structures during development and across the lifespan (Heim et al., 2019). The PFC and amygdala exert, respectively, inhibitory and stimulatory effects on hypothalamic CRH neurons via indirect projections (Herman et al., 2020) and, hence, the constellation of ELS-related neural alterations appears to promote stress responses (Figure 2). Decreased structural and functional connectivity between the medial PFC and the amygdala are reported after ELS, and this was predicted by cortisol levels at the age of 4.5 years (Burghy et al., 2012). The amygdala-PFC circuit, implicated in threat-reactivity and emotion regulation, is particularly sensitive to environmental inputs, especially during early life leading to a loss of “top down” control of emotional responses, fear learning, and stress responses, which may converge into heightened disease risk (VanTieghem and Tottenham, 2018). The amygdala is particularly sensitive to the effects of abuse at the age of around 10 years, whereas the hippocampus has heightened sensitivity at an earlier age, and the PFC seems to be particularly amenable around puberty (Teicher et al., 2016).

Gender-specific differences in brain regions occur mainly in areas containing sex steroid receptors such as the hypothalamus or in regions closely related to areas with high density of sex steroid receptors such as the amygdala (Lenroot and Giedd, 2010; Kaczkurkin et al., 2019), which plays a central role in negative emotion and threat processing (LeDoux, 1992). Sex is a prominent source of variability for HPA axis stress responses (Kudielka and Wüst, 2010). Developmental trajectories differ for men and women thus ELS occurring at the same time can lead to diverse and gender-specific outcomes.

### Early-life stress and inflammatory bowel disease

Agrawal et al. (2021) systematically reviewed the impact of pre-, peri-, and postnatal exposures up to the age of five years on subsequent IBD diagnosis. They concluded that early life is an important period of susceptibility for IBD development later in life. Tobacco smoke, infections and antibiotics were associated positively, while breastfeeding was associated negatively with IBD. However, ELS was not evaluated in their study which was limited to the age of five years while, as stated above, the amygdala is particularly sensitive to the effects of abuse at the age of around 10 years, and the PFC seems to be particularly amenable around puberty (Teicher et al., 2016).

In a retrospective cohort design, based on the Manitoba IBD cohort, Witges et al. (2019) reported that almost 74.2% of their cohort of IBD patients was exposed to one or more ELS. Most of the patients (51.3%) reported death of a family member during childhood, 12.2% reporting physical abuse, and 13% sexual abuse, while upheaval between parents was experienced by one fifth (20.3%) of participants. Emotional abuse was not specified in this study. These data are higher than previously reported in other US and Canadian population-based studies (55 to 60% of exposed IBD patients) (Metzler et al., 2017; McDonald et al., 2015). After consideration of other covariates, any reported ELS was not predictive of increased non-IBD-related or IBD-related healthcare. However, the authors estimated that ELS may still have a significant impact on persons with IBD since IBD patients exposed to physical abuse, sexual abuse, and parental upheavals were utilizing more healthcare than non-exposed IBD patients. The authors argue that clinicians should consider inquiry into ELS as a component of IBD care to better counsel their IBD patients in terms of managing psychosocial factors, including stress, anxiety, and depression, and seeking out the appropriate supports (Graff et al., 2009).

Exposure to an ELS can serve as an important environmental trigger or risk factor for IBD outbreak (Bernstein et al., 2010). In a national register-based cohort study of men in Sweden followed from late adolescence to middle age, Melinder et al. (2017) showed that lower stress resilience in adolescence was associated with increased IBD risk for CD and UC (after adjustment with subclinical disease activity in adolescence). Atanasova et al. (2021) did not report higher levels of adverse childhood experiences in their IBD sample compared to healthy controls but patients reporting current and/or lifetime psychiatric disorders were not included in their study.

We also recently evaluated the prevalence of ELS in a sample of 93 IBD patients, prospectively recruited, and estimated the burden of these early abuses on their mental, GI and physical health states (Minjoz et al., 2023). A total of 53% of participants reported being exposed to childhood abuse, of whom 49% were physically abused, 87.8% emotionally abused, and 20.4% sexually abused. Among the 49 patients exposed to childhood abuse, 27 (55.1%) reported having been exposed to just one type of abuse (22 emotional, 3 physical, 2 sexual), 16 (32.7%) reported two types of abuse (14 emotional and physical, one sexual and emotional, one sexual and physical), and 6 (12.2%) reported three types of abuse (emotional, physical, and sexual). Patients who were exposed to ELS, compared with those who were not, reported that they were strongly more devaluated (not feeling like an equal member of the family) and threatened (threats and punishments from one’s parents), and had submitted moderately more (e.g., feeling nervous for fear of parental anger), referring to more affective deprivation. Emotional abuse was the most predominant (87.8%), in agreement with the larger prevalence of emotional abuse in ELS studies compared with physical and sexual abuse (Gama et al., 2021). Emotional abuse can coexist with physical and/or sexual abuse within the same abusive relationship (Geffner and Rossman, 1997), and emotional abuse includes various forms of psychological maltreatment, trauma, and non-physical aggression (Thompson and Kaplan, 1996). Emotional status can negatively affect disease activity and quality of life, and increases the suicide risk (Tripp et al., 2022). Mental health disorders (perceived-stress, anxiety, depression, emotion-centered coping) were significantly higher in our patients exposed to childhood abuses than unexposed. Exposed patients had also lower GI quality of life associated with more digestive perturbations (abdominal pain and discomfort, change in stool consistency, flatulence) and more fatigue. Finally, no significant difference in visiting medical frequency, other digestive disturbances (stool frequency, heartburn, early satiety, nausea, and vomiting), extra-digestive pain (joint, muscle, headache, diffuse pain, low back pain) and other health complaints (sleep, fever, urinary disorders, lethargy) were observed in our study.

In another study, Gnat et al. (2023) recruited 195 IBD patients and 190 healthy controls through online communication platforms (e.g., Facebook groups and Instagram). IBD patients reported more sexual, disruptive, and violent traumas. Although confiding did not act as a moderator, trauma was related to depressive symptoms through resilience. It should be noted that the healthy controls and patients in the samples differed significantly in age, gender, race, and country of residence.

Although the prevalence of ELS does not appear systematically higher in IBD than in healthy volunteers (at least without IBD), on the other hand, in IBD patients, having experienced emotional abuse of any type (the main dimension of ELS) worsens the experience of the disease and further impairs their quality of life. In addition, ELS should be considered in a genetic and epigenetic (particularly DNA methylation) context (Fogelman and Canli, 2019) and there is a genetic susceptibility in IBD patients.

The difference between prevalences reported for ELS in the study of Witges and ours is quite large (74.2 versus 53%) but our patients were prospectively included while a retrospective cohort design was adopted in the study of Witges et al. (2019). In addition, when measuring events that may have occurred years earlier, it is possible that patients were unable to recall exposure or the associated trauma, or they could have had false memories. Patient interviews and self-reports have limitations. Memories of remote and early traumatic experiences are often suppressed or erased. Moreover, identifying participants whose responses are likely dishonest or questionable is challenging. Documentation requires objectivity and the value of an interview remains restricted. Even when equipment and staffing are adequate, different issues (for example, retraumatization) might lead to the loss of information (Vyshka et al., 2023).

Taken together, these results suggest that ELS should be considered as a component of IBD care because adverse childhood experiences can serve as an aggravating factor for IBD and exacerbate symptoms related to it. This could lead to better counsel IBD patients and seeking out the appropriate supports in the perspective of a personalized holistic care. As corroborating arguments, recent studies point out the link between ELS and chronic diseases that involve an inflammatory background such as obesity, diabetes and cardiovascular diseases (Godoy et al., 2021; Koball et al., 2024; Zhu et al., 2022). Indeed, inflammatory cytokines, such as TNF, are much more elevated during mid-life in patients reporting history of physical or emotional abuse than patients with no ELS. Hence, inflammatory cytokines could be risk factors for chronic disease development and immune system dysregulation as a common mediating process (Furman et al., 2019). At that time, no similar data are available in IBD. However, some studies suggest this link through the mediation of depression. Indeed, people with ELS are at risk of both increased inflammation and depression in later life (Danese et al., 2009). Childhood trauma is a common and potent risk factor for developing major depressive disorder in adulthood, associated with earlier onset, more chronic or recurrent symptoms, and greater probability of having comorbidities (Childhood Trauma Meta-Analysis Study Group, 2022; Petruccelli et al., 2019). In addition, there is a link between depression and inflammation as a vicious circle: depression promotes inflammation and vice-versa. Depressive symptoms occur in more than 20% of IBD patients, which is ∼ two to four times more common than in the general population (Neuendorf et al., 2016; Bisgaard et al., 2022). There are increased risks of depression and anxiety both before and after IBD diagnosis (Bisgaard et al., 2022). In turn, depressive symptoms are also associated with T cell activation and proinflammatory cytokines (Beurel et al., 2013; Martin-Subero et al., 2016). Depressive symptoms are a further extra-intestinal manifestation of inflammation in IBD patients (Moulton et al., 2020). Patients with depression increase the risk of IBD, which may be mitigated by the use of anti-depressants (Frolkis et al., 2019). We have also reported depression in a population of IBD patients even in remission (Pellissier et al., 2014). The co-morbidity of depressive symptoms and IBD is associated with poor biomedical outcomes, such as increased risk of IBD relapse, hospitalization, requirement for biologics and surgery i.e., more aggressive IBD (Ananthakrishnan et al., 2013; Kochar et al., 2018; Fairbrass et al., 2023). Blackwell et al. (2021) have recently shown that IBD patients have a higher prevalence of depression than matched controls as early as 9 years before diagnosis. Depression in the absence of prior GI symptoms was not associated with a future diagnosis of IBD but those with depression diagnosed after already experiencing GI symptoms are at increased risk of later being diagnosed with IBD. Somatic symptoms associated with depression, such as fatigue and sleep disturbance, are more strongly associated with elevated inflammation than more cognitive symptoms, such as low mood and guilt (Duivis et al., 2015; Loftis et al., 2013).

In conclusion, ELS, stress, anxiety, and depression should be considered as components of IBD care and should be systematically sought and evaluated. This could allow a better management of IBD patients and offer them the appropriate drug- or non-drug therapeutic supports in the perspective of a personalized holistic care.

### Potential mechanisms linking childhood early-life stress and inflammation

**FIGURE 3:**
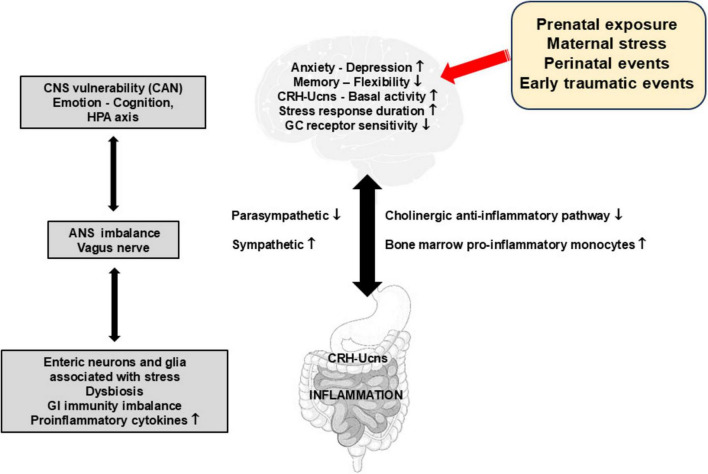
Impact of early stressful and traumatic events on the function of the gut-brain axis and the occurrence of gut inflammation. This figure presents the multilevel effects of stress and early traumatic events on various physiological and psychological aspects. These include an increase in basal CRH-Ucn release, pro-inflammatory responses via the recruitment of pro-inflammatory phenotype cells in bone marrow but also in the enteric nervous system (neurons and glia) and sympathetic activity, as well as a decrease in memory flexibility, GC receptor sensitivity and parasympathetic activity. These perturbances create imbalances in the autonomic nervous system and in gastrointestinal immunity, underscoring the susceptibility of the central nervous system and the incidences for emotional regulation, cognitive function, and the hypothalamic-pituitary adrenal axis. ANS, autonomic nervous system; CAN, central autonomic network; CNS, central nervous system; CRH-Ucns, corticotrophin-releasing hormone-Urocortins; GC, glucocorticoids; HPA, hypothalamic-pituitary adrenal; GI, gastrointestinal.

At the pathophysiological level (Figure 3), ELS would induce hyperactivation of the central noradrenergic and CRHergic systems, and possibly of the GI CRHergic system, leading to dysfunction of the neuro-endocrine-immune axis and the sympatho-adrenergic axis, as well as low vagal tone i.e., a proinflammatory state thus promoting subsequent IBD in genetically susceptible patients (Bonaz and Bernstein, 2013; Bonaz et al., 2017; Labanski et al., 2020). There is emerging evidence for a role of the SNS as one of the main mechanisms behind the link between early life adversities and peripheral or central inflammation. The SNS appears to play a central role by increasing the activation of T cells and shifting the differentiation of hematopoietic stem cells in the bone marrow to proinflammatory monocytes, with elevated expression of proinflammatory molecules (Mondelli and Vernon, 2019). Preclinical studies in adult animals suggest that trafficking of peripheral immune cells to the brain is an important mechanism for the development of anxiety following stress exposure (Mondelli and Vernon, 2019). There is an important lack of studies addressing these issues in human.

There is a link between negative affect, vagal tone, and visceral sensitivity in IBD (Rubio et al., 2014). There is also, in some IBD patients in remission, a defect of vagal tone coupled to higher threshold of TNF plasma level thus creating a real state of vulnerability to flare (Pellissier et al., 2014). As a matter of fact, we can hypothesize that high perceived stress activates the SNS and inhibits the VN thus favoring inflammation through the induction of proinflammatory macrophage precursors (sympathetic arm) and the inhibition of the CAP (parasympathetic arm).

The brain and the immune system are not fully formed at birth, but rather keep on maturing in response to the postnatal environment. The two-way interaction between the brain and the immune system makes it possible for childhood psychosocial stressors to affect immune system development, which in turn can affect brain development and its long-term functioning. Drawing from experimental models and observational human studies, Danese and Lewis (2017) propose that the psychoneuroimmunology of ELS can offer an innovative framework to understand and treat psychopathology linked to childhood trauma and stressful events as they predict later inflammation by the striking analogies between the neurobiological correlates of ELS and those of inflammation.

Psychological trauma may occur in the context of physical trauma. In this case, physical injury and pathogen infection can induce inflammation by triggering innate immunity (Medzhitov and Janeway, 2000). Short-term activation of the inflammatory response during sensitive periods in early life may affect the brain development and later microglia and neuroendocrine reactivity. ELS may be indirectly linked to inflammation because of primary neuroendocrine abnormalities in the HPA axis. Childhood trauma is associated with later hyperactive HPA axis functioning (Danese and McEwen, 2012), presumably because of primary abnormalities of the GC receptor and then an attenuation of the negative feedback loop.

Both preclinical and clinical studies have shown that ELS is associated with epigenetic changes, leading to impaired functioning of the GC-receptor-mediated signaling (Zannas and Chrousos, 2017). These changes could in turn induce resistance to the anti-inflammatory properties of cortisol and, thus, high inflammation levels (Raison and Miller, 2003). However, because of the bi-directional association between HPA axis functioning and inflammation, it is also possible that primary inflammatory abnormalities could stimulate HPA axis activity (Besedovsky et al., 1986) and induce GC resistance (Barnes and Adcock, 2009). Second, ELS may influence the composition of the gut microbiota (Hantsoo and Zemel, 2021; Petitfils et al., 2023). Indeed, early-life adversity can influence the development of the gut microbiome and potentially make more prone to various diseases (Charalambous et al., 2021). In turn, gut dysbiosis could influence brain function through the VN and other metabolic effects (Bonaz et al., 2018). Third, ELS is associated with hormonal and brain abnormalities that could contribute to a “thrifty” phenotype characterized by increased energy intake and storage, and/or reduced energy expenditure resulting in obesity (Danese and Tan, 2014). In turn, obesity is associated with high systemic inflammation through the production of pro-inflammatory cytokines by adipocytes (Gregor and Hotamisligil, 2011). In an adult population, obesity as measured by body mass index, was associated with an increased risk of older-onset CD but not UC (Chan et al., 2022). Fourth, ELS has been linked to alcohol and substance abuse disorders and smoking (Dube et al., 2003; Anda et al., 1999), which can increase inflammation levels (Crews et al., 2006; Shiels et al., 2014). Fifth, ELS has been associated with decreased total sleep and disruption in sleep architecture in rodents (Feng et al., 2007; Mrdalj et al., 2013; Tiba et al., 2004) and humans (Gregory and Sadeh, 2016; Kajeepeta et al., 2015). Chronic sleep disturbance results in HPA axis and SNS activation, both of which upregulate a pro-inflammatory immune response, leading to an increase in systemic inflammatory activity (Irwin, 2015). There is a positive association between short sleep duration (≤ 5 h/day), and daytime napping, with later development of incident IBD (Yuan et al., 2023). Finally, ELS is associated with later abnormalities in brain functioning and behavior (Herzberg and Gunnar, 2020). Persistent or recurrent distress and self-harming behaviors described in individuals with a history of childhood trauma could contribute to maintaining elevated inflammation levels (Baumeister et al., 2016).

Very recently, Schneider et al. (2023) discovered a critical role for the enteric nervous system in mediating the aggravating effect of chronic stress on intestinal inflammation in mouse models of IBD. They found that chronically elevated levels of GC drive the generation of an inflammatory subset of enteric glia that promotes monocyte- and TNF-mediated inflammation via colony-stimulating factor 1. Blocking colony-stimulating factor 1 signaling prevented the exacerbating effect of stress on intestinal inflammation. In addition, stress and GC caused a transforming growth factor β2-mediated shift toward an immature enteric-neuron phenotype, neuron loss, and gut dysmotility. Data from the UK Biobank and independent cohorts of IBD patients also indicated that chronic psychological stress is associated with gut dysmotility, higher levels of intestinal inflammation, and greater IBD severity (Schneider et al., 2023). Together, these findings offer a mechanistic explanation for the impact of the brain on peripheral inflammation, define the enteric nervous system as a relay between psychological stress and gut inflammation, and suggest that stress management could serve as a valuable component of IBD care.

## Animal models to demonstrate cellular and molecular mechanisms of the relation between early-life stress and gastrointestinal inflammation

Zhong et al. (2018) used a 2-hit rat model to induce neonatal and adult inflammation. They showed that neonatal inflammation sensitizes the colonic epithelium for exacerbated interleukin (IL)1β activation by increasing stress hormones that induce histone hyperacetylation, thus allowing greater access of nuclear factor-κB to the IL1B promoter and rendering the host susceptible to aggravated immune responses. The inflammatory response and IL1β overexpression were markedly improved by Propranolol, a β-blocker, by mitigating against epigenetic modifications thus suggesting that β-blockers have a therapeutic potential for IBD susceptibility, and establishing a novel paradigm whereby neonatal inflammation induces epigenetic susceptibility to IBD.

Kline et al. (2020) used the same “double-hit” rat model to identify disease mechanisms associated with ELS that exacerbate IBD in adulthood. They observed a sustained increase in colonic permeability in these rats with a significantly decreased expression of the epithelial junction protein E-cadherin. They found an overexpression of microRNA-155, a predicted down regulator of E-cadherin expression. Rectal administration of a microRNA -155 inhibitor significantly reversed colonic permeability. Thus, early-life inflammatory stressors trigger a significant and sustained epithelial injury by suppressing E-cadherin through epigenetic mechanisms. We have shown that CRH2 signaling could modulate intestinal epithelial cell differentiation (Ducarouge et al., 2017). Indeed, Urocortin 3, a selective CRH2 agonist, alters both para- and trans-cellular permeability of differentiated HT-29 and Caco-2 cells. Urocortin 3-mediated activation of CRH2 decreases mRNA and protein expression levels of KLF4, a transcription factor involved in intestinal epithelial cell differentiation. This signaling was correlated to a down-regulation of key intestinal epithelial cell markers such as the dipeptidyl peptidase IV and alkaline phosphatase enzymes, at both transcriptional and post-transcriptional levels (Ducarouge et al., 2017). These mechanisms could be relevant to the stress-induced epithelial alterations found in IBD.

Very recently, Muir et al. (2023) used two published animal models of ELS in mice (neonatal maternal separation and postnatal dexamethasone exposure). Colitis was induced in 4-week-old mice via intraperitoneal injection of IL-10 receptor blocking antibody every 5 days for 15 days. ELS resulted in HPA axis dysfunction with reduced circulating levels of corticosterone and impaired production of corticosterone in the colon, known to preserve the epithelial barrier, both in the adolescent period shortly after removal of the stress signal and in adulthood. This reduced local corticosterone may be central to the increased inflammatory tone of the intestine, characterized by early deficits in mucosal IL-10–producing CD4 T cells followed by increased interferon γ-producing CD4 T cells in early adulthood. Thus, an environmental trigger, as represented by a colitogenic insult (antibody-mediated blockade of the IL-10 receptor), can induce and imprint disruptions in normal gut immune homeostasis and can have long-term effects on the predisposition to and perpetuation of chronic inflammation in susceptible hosts.

## Therapeutic perspectives and conclusion

Based on neuroanatomical and neurofunctional data, there is growing evidence that ELS can favor and/or aggravate, later in life, IBD which is a bio-psycho-social model with disorder of gut-brain interactions. Perturbations of the HPA axis, the CRHergic system, and the ANS can induce GI motility disturbances, intestinal permeability increase, dysbiosis, visceral hypersensitivity, and pro-inflammatory conditions contributing to the development/and or worsening of IBD.

There are presently unmet needs of pharmacological treatments in the management of IBD (Bonaz, 2024). Indeed, treatments are not devoid of side effects, cost-effective, do not cure the disease, can lose effects over time, thus explaining the poor patient satisfaction, their lack of compliance, and their interest for non-drug therapies. The gut-brain axis can be targeted for therapeutic purposes in IBD through non-drug therapies. Targeting pathways, such as the CAP, which are involved in such pro-inflammatory conditions, are of interest. Vagus nerve stimulation (VNS, i.e., Bioelectronic Medicine) can improve active CD, restore a homeostatic vagal tone and redirect pro-inflammatory cytokines and metabolomics to healthy profiles (Bonaz et al., 2016; Sinniger et al., 2020; D’Haens et al., 2023). Transcutaneous auricular VNS targets the same central pathways than invasive cervical VNS, is easier to perform, without side effects or off target effects of invasive cervical VNS, and has anti-inflammatory properties (Drewes et al., 2021; Wu et al., 2023). In addition, transcutaneous auricular VNS significantly increases amygdala-dorsolateral PFC connectivity, thus restoring an equilibrium between the ANS and the HPA axis (Liu et al., 2020). Non-invasive transcutaneous auricular VNS in healthy volunteers consistently reduces the permeability of the small intestine (Mogilevski et al., 2022). Such an effect on intestinal permeability could participate to the positive effect of VNS reported in CD patients. Indeed, the VN can modulate the permeability of the intestinal barrier, although it does not innervate directly the intestinal epithelium, through enteric nerves, and/or cells such as enteric glial cells, and there is evidence of VNS effects on intestinal permeability in models such as burn intestinal injury and traumatic brain injury (Bonaz, 2022). Pharmacologic manipulation of the CAP is also of interest using selective alpha7-nicotinic agonists or central cholinergic activation induced by the acetylcholinesterase inhibitor galantamine alleviated an experimental colitis (Ji et al., 2014), anti-depressants (see above), systemic anti-inflammatory treatments such as cytokine antagonists (anti-TNF, anti-IL12/23), and anti-integrins. One can imagine that bioelectronic anti-inflammatory approaches targeting the brainstem dorsal motor nucleus of the VN through deep brain stimulation, could stimulate the CAP, as reported in mice alleviating lipopolysaccharide-induced inflammation (Falvey et al., 2024). Along the same lines, based on the role of the insula in the control of peripheral immunity (Koren et al., 2021), deep brain stimulation or transcranial alternating current stimulation of the insula, as performed in pain or neuropsychiatric conditions (Galhardoni et al., 2019; Shan et al., 2023), could be of interest. Both IBD and mental disorders are associated with gut microbial alterations. Targeting the gut microbiota may represent a useful therapeutic approach for the treatment of psychiatric co-morbidities in IBD (Thomann et al., 2020). Therapies such as cognitive behavioral therapies and hypnosis are recommended in the management of stress reduction in IBS (Chey et al., 2021). Gut-directed hypnosis is well known to improve IBS patients with a prolonged effect over time (Gonsalkorale and Whorwell, 2005; Flik et al., 2019) and is recommended by the American Gastroenterological Association. Cognitive behavioral therapies-based interventions and gut-directed hypnotherapy had the largest evidence base and were the most efficacious in the long term (Black et al., 2020). Gut-directed hypnotherapy significantly prolongs clinical remission in quiescent UC (Keefer et al., 2013) and is efficient for IBS-type symptoms in IBD patients in remission (Hoekman et al., 2021). In a pilot study performed in IBD patients, we reported that gut-directed hypnosis has a positive effect on short-term quality of life, clinical symptoms, disease acceptability, perception of disease benefits and physiological stress (Artru-Voegelé et al., 2022). A recent systematic review and meta-analysis showed that interventions including cognitive behavioral therapies, mindfulness-based therapy, breath-body-mind-workshop, guided imagery with relaxation, solution-focused therapy, yoga, and multicomponent interventions significantly helped to reduce anxiety, depression, and disease specific quality of life in IBD adults compared to control groups (Davis et al., 2020a), and are helpful in managing IBD-fatigue (Davis et al., 2020b). However, the effect sizes are small. No firm conclusions regarding the efficacy and safety of non-pharmacological interventions can be drawn on the management of fatigue in IBD (Farrell et al., 2020). Mindfulness interventions are effective in reducing stress and depression and improving quality of life and anxiety, but do not lead to significant improvements in the physical symptoms of IBD (Ewais et al., 2019).

In a systematic search, Paulides et al. (2021) reported that psychotherapeutic interventions can improve quality of life in IBD patients. Treatment modalities differed in the reported studies and consisted of cognitive-behavioral therapy, psychodynamic therapy, acceptance and commitment therapy, stress management programs, mindfulness, hypnosis, or solution-focused therapy. All four studies focusing on patients with active disease reported a positive effect of psychotherapy on quality of life of IBD patients. Trials applying cognitive-behavioral therapy reported the most consistent positive results. In a small pilot study in 16 adolescents with ELS, there was some evidence for efficacy of mindfulness-based stress reduction on a symptom level (depressive) with potential subtle changes on a biological level (immune and endocrine biomarkers) although future larger studies are needed (Cohen et al., 2021). In a recent meta-analysis including 25 randomized controlled trials, Riggott et al. (2023) reported that psychological therapies have beneficial, short-term effects on anxiety, depression, stress, and quality-of-life scores, but not on disease activity. The authors concluded that further trials in selected groups are needed to establish the place for such therapies in IBD care. A very recent systematic review and meta-analysis (28 randomized controlled trials involving 1,789 participants) reported that treatments which address mood outcomes have beneficial effects on generic inflammation as well as disease-specific biomarkers (fecal calprotectin and C-reactive protein). Psychological interventions and interventions with larger treatment effects on mood accentuated the effect on biomarkers (Seaton et al., 2024). However, thirty-eight percent of patients reported using psychotherapy to address IBD-specific issues (Craven et al., 2019). IBD patients want psychosocial issues to be addressed but patient perceptions that there are insufficient mental health providers who are knowledgeable about IBD, and the greatest endorsed barrier to psychotherapy was cost (Craven et al., 2019).

Physical exercise has an anti-inflammatory effect through an activation of the VN (Laborde et al., 2018). Jones et al. (2022) have reported that structured exercise programmes improve disease activity, but not disease-specific quality of life in IBD patients. Defining an optimal exercise prescription and synthesis of evidence in other outcomes, was limited by insufficient well-designed studies to ascertain the true effect of exercise training. This warrants further large-scale randomized trials employing standard exercise prescription to verify this effect to enable the implementation into clinical practice.

Clinicians should be aware of techniques for potentially modifying early life factors including early detection for promoting symptom management with cognitive therapies and parental education about social learning of illness behavior in children. Prevention interventions to reduce the associated psychological impact should be part of the treatment plan for IBD patients. Taking into account ELS could allow identifying subgroups of patients who may be more vulnerable and therefore, optimize IBD management and prevent the worsening of the disease. Further studies that deeply investigate ELS and its link with negative health outcomes are also warranted in IBD patients. To our knowledge, there is presently no data on the prevention of IBD by early detection and management of ELS.

Therefore, any therapy, whether drug-based or not, capable of restoring the balance of vagal activity and that of the HPA axis has a potential interest in IBD. These results may impact counseling and management of IBD patients.
